# Lifting the Veil: Delayed Diagnosis of Sheehan Syndrome Unmasked by Adrenal Crisis

**DOI:** 10.1210/jcemcr/luaf190

**Published:** 2025-08-29

**Authors:** Amanda Alkhafaji, Dhruva Govil, Frank Shaya, Ahmad Ather

**Affiliations:** Henry Ford Providence Southfield Internal Medicine, Southfield, MI 48075, USA; Henry Ford Providence Southfield Internal Medicine, Southfield, MI 48075, USA; Henry Ford Providence Southfield Internal Medicine, Southfield, MI 48075, USA; Henry Ford Providence Southfield Internal Medicine, Southfield, MI 48075, USA

**Keywords:** Sheehan syndrome, adrenal insufficiency, hypothyroidism, case report

## Abstract

We report a 28-year-old woman with refractory hypoglycemia, hypotension, and profound fatigue found to have panhypopituitarism secondary to Sheehan syndrome. Although she had a remote history of postpartum hemorrhage marked by agalactia and secondary amenorrhea, her diagnosis was delayed until she developed an adrenal crisis in the setting of acute pyelonephritis. Comprehensive endocrine testing confirmed secondary adrenal insufficiency, central hypothyroidism, hypogonadotropic hypogonadism, and lactotroph failure; Magnetic resonance imaging demonstrated a partially empty sella consistent with remote pituitary infarction. Prompt initiation of stress-dose glucocorticoids and thyroid hormone led to rapid hemodynamic stabilization and resolution of hypoglycemia. This case underscores the importance of early recognition of subtle hypopituitarism signs—particularly postpartum lactation failure—and the need to consider endocrine etiologies in critical care presentations that mimic septic shock.

## Introduction

Panhypopituitarism denotes a deficiency of all anterior pituitary hormones. Sheehan syndrome, ischemic necrosis of the pituitary following severe postpartum hemorrhage, remains uncommon in high-resource settings but may present insidiously years after the inciting event. Acute decompensation often occurs only under physiologic stress, when cortisol deficiency precipitates shock and hypoglycemia that can be misattributed to sepsis. Early recognition and hormone replacement are essential to prevent life-threatening crises.

## Case Presentation

A 28-year-old woman with a history of asthma and recently diagnosed, untreated central hypothyroidism (identified postpartum) who presented with 4 days of nausea, bilious vomiting, diarrhea, profound fatigue, cold intolerance, and an unintentional 22.6 kg weight loss over the past year. She also reported multiple episodes of documented hypoglycemia in the outpatient setting. Of note, she recalled failure to lactate following a complicated vaginal delivery 14 months prior, which was notable for placental abruption and acute blood loss requiring transfusion. This was followed by irregular menses, though she did not pursue further evaluation, including imaging and labs.

On arrival, her vital signs revealed hypotension (blood pressure 82/50 mmHg), tachycardia (heart rate 110 bpm), and severe hypoglycemia with a capillary glucose 28 mg/dL (SI: 1.6 mmol/L) (reference range, 70-99 mg/dL [SI: 3.9-5.5 mmol/L]). She appeared cachectic and lethargic, without focal neurologic deficits or visual complaints.

## Diagnostic Assessment

The patient's biochemical profile revealed significant hyponatremia of Na⁺ 128 mmol/L (SI: 128 mEq/L) (reference range, 135-145 mEq/L [SI: 135-145 mmol/L]), mild hypokalemia of 3.6 mEq/L (SI: 3.6 mmol/L) (reference range, 3.5-5.0 mEq/L [SI: 3.5-5.0 mmol/L]), a low bicarbonate level of 14 mEq/L (SI: 14 mmol/L) (reference range, 22-28 mEq/L [SI: 22-28 mmol/L]), and an elevated anion gap of 18, consistent with a high anion gap metabolic acidosis. Serum creatinine was mildly elevated at 1.5 mg/dL (SI: 132.6 μmol/L) (reference range, 0.6-1.2 mg/dL [SI: 53-106 μmol/L]), and lactate was markedly elevated at 6.2 mmol/L (reference range, 0.5-2.2 mmol/L), suggesting tissue hypoperfusion or sepsis. Hypoglycemia was noted with a glucose level of 28 mg/dL (SI: 1.6 mmol/L).

Imaging studies including a computed tomography abdomen demonstrated right-sided pyelonephritis with multiple 1.2 cm microabscesses, as well as small pleural effusion and ascites. A right upper quadrant ultrasound showed cholelithiasis with complex pericholecystic fluid, raising concern for possible evolving cholecystitis. Blood and urine cultures returned negative. An endocrine evaluation revealed marked adrenal insufficiency, with a low morning cortisol level of 2.9 mcg/dL (SI: 80 nmol/L) (reference range, 6-18 µg/dL [SI: 165-497 nmol/L]) and suppressed ACTH of <3 pg/mL (SI: <0.66 pmol/L) (reference range, 10-60 pg/mL [SI: 2.2-13.2 pmol/L]). Central hypothyroidism was indicated by low free T4 of 0.27 ng/dL (3.48 pmol/L) (reference range, 0.8-1.8 ng/dL [SI: 10-23 pmol/L]) with inappropriately low to normal TSH 3.5 mIU/L (reference range, 0.4-4.0 µIU/mL). Prolactin was undetectable at 0.3 ng/mL (SI: 6.4 mIU/L) (reference range, 4.8-23.3 ng/mL [SI: 102-496 mIU/L]). Her hormone levels were drawn during the follicular phase of the menstrual cycle. Estradiol was within range at 48 pg/mL (176 pmol/L) (reference range, 15-350 pg/mL [SI: 55-1285 pmol/L]); however, FSH and LH were inappropriately normal, suggesting central hypogonadism. IGF-1 was also below the age-matched reference range. Aldosterone was decreased at 1.0 ng/dL (SI: 27.7 pmol/L) (reference range, 4-31 ng/dL [SI: 110-860 pmol/L]). Magnetic resonance imaging of the pituitary showed a 6 mm T1 hyperintense focus with a partially empty sella, consistent with prior pituitary infarction. Altogether, these findings are consistent with panhypopituitarism, likely due to remote pituitary infarction, contributing to the patient's hypoglycemia, adrenal insufficiency, and central hypothyroidism.

## Treatment

Once in the intensive care unit, the patient was initiated on stress-dose hydrocortisone at 100 mg IV every 12 hours and received an IV 200 mcg loading dose of levothyroxine, followed by 88 mcg orally each day. Supportive management included IV fluids, dextrose infusion, and norepinephrine for refractory hypotension. Within 24 hours, her hemodynamics stabilized, blood glucose normalized without the need for ongoing dextrose infusion, and serum lactate levels decreased to within normal limits. Once the patient stabilized off pressor support, she was transferred to the floor where she completed a 10-day course of IV ertapenem for pyelonephritis. Upon stabilization, she was transitioned to maintenance therapy with hydrocortisone 15 mg in the morning and 10 mg in the evening, along with continued daily oral levothyroxine at 88 mcg.

## Outcome and Follow-up

The patient was discharged from the hospital with close follow-up with an endocrinologist and a primary care doctor. She was instructed that her outpatient follow-up would consist of gonadal hormone evaluation and replacement, GH axis assessment, bone density scan, and education on stress dosing and adrenal crisis management.

## Discussion

This case exemplifies the chronic, indolent course of Sheehan syndrome in a developed care setting, where postpartum agalactia and amenorrhea went uninvestigated until an acute stressor precipitated an adrenal crisis. Similar cases in the literature often report Sheehan syndrome presenting years after the obstetric insult, with nonspecific features—fatigue, weight loss, cold intolerance—until secondary adrenal insufficiency and central hypothyroidism are unmasked under stress, underlying the major limitation in management here [1]. Clinical guidelines from the Endocrine Society stress the importance of immediate glucocorticoid replacement during adrenal crisis, followed by a comprehensive pituitary evaluation, including cortisol, ACTH, thyroid function tests, prolactin, gonadotropins, and imaging, to establish the diagnosis and guide long-term hormone replacement therapy [2]. The magnetic resonance imaging findings of a partially empty sella are particularly characteristic of this remote pituitary infarction [3, 4].

These patients require lifelong, multidisciplinary care to optimize hormone replacement, monitor bone health, and educate on sick day rules. Early diagnosis can prevent repeated intensive care unit admissions and significantly improve quality of life. Sheehan syndrome remains an important but often overlooked cause of panhypopituitarism. Recognition of subtle postpartum clues and prompt management of endocrine emergencies can avert potentially fatal crises [5]. Clinicians should maintain a low threshold for pituitary evaluation in women with unexplained hypoglycemia, hypotension, and chronic fatigue following complicated childbirth.

## Learning Points

- Sheehan syndrome should be considered in women with an obstetric hemorrhage history and postpartum lactation failure.
- In adrenal crisis, immediate stress-dose glucocorticoids are lifesaving and should precede thyroid replacement to avoid precipitating further adrenal insufficiency.
- A comprehensive evaluation of all pituitary axes is essential; low prolactin in the postpartum period, inappropriately normal gonadotropins, and low IGF-1 all point to panhypopituitarism.
- Differentiation from pituitary apoplexy hinges on obstetric history and lack of acute neurologic signs (eg, severe headache, visual field defects).

## Contributors

All authors made individual contributions to authorship. D.G., A.A., F.S., and A.A. were involved in the diagnosis and management of this patient. All authors reviewed and approved the final draft.

## Data Availability

Data sharing is not applicable to this article as no datasets were generated or analyzed during the current study.
